# The Amount of the Rare Sugar Tagatose on Tomato Leaves Decreases after Spray Application under Greenhouse Conditions

**DOI:** 10.3390/plants11202781

**Published:** 2022-10-20

**Authors:** Abdessalem Chahed, Andrea Nesler, Qassim Esmaeel, Essaid Ait Barka, Michele Perazzolli

**Affiliations:** 1 Research and Innovation Centre, Fondazione Edmund Mach, Via E. Mach 1, 38098 San Michele all’Adige, Italy; 2 Bi-PA nv, Technologielaan 7, 1840 Londerzeel, Belgium; 3 Induced Resistance and Plant Bioprotection, USC INRAE 1488, University of Reims, UFR Sciences, CEDEX 02, 51687 Reims, France; 4 Center Agriculture Food Environment (C3A), University of Trento, Via E. Mach 1, 38098 San Michele all’Adige, Italy

**Keywords:** rare sugar, *Phytophthora infestans*, phytopathogenic oomycete, disease control, tomato

## Abstract

Tagatose is a rare sugar that suppresses plant diseases, such as late blight of tomato, caused by *Phytophthora infestans*. Tagatose can be metabolized by some microorganisms and no information is available on its persistence on tomato leaves. The aim of this study was to assess the persistence of tagatose on tomato leaves under commercial greenhouse conditions. The amount of tagatose on tomato leaves and the inhibitory activity against *P. infestans* decreased seven days after spray application in the absence of rain wash-off. Potential tagatose-degrading bacteria were isolated from tomato leaves, and they belonged to *Acinetobacter* sp., *Bacillus* sp., *Comamonas* sp., *Enterobacter* sp., *Methylobacterium* sp., *Microbacterium* sp., *Pantoea* sp., *Plantibacter* sp., *Pseudomonas* sp., *Ralstonia* sp., *Rhodococcus* sp., *Sphingobium* sp., and *Sphingomonas* sp. Thus, indigenous phyllosphere microorganisms could partially metabolize tagatose laid on plant leaves after spray application, reducing the persistence of this fungal inhibitor on tomato leaves.

## 1. Introduction

Rare sugars have been defined as monosaccharides and their derivatives that rarely exist in nature [1]. The biological properties of rare sugars are not fully understood, and their promising applicative values are underestimated, mainly because of their limited available quantity in nature [2,3]. However, the implementation of novel enzymatic and microbial processes lowered the cost of rare sugar synthesis and extended their use in several scientific and technological areas of agriculture, human nutrition, and medicine [2,4]. Among rare sugars, tagatose is a ketohexose that was found at low concentrations within various food products, such as apples, oranges, milk, and cheese [5]. Tagatose is naturally present as a metabolic intermediate of the tagatose-6-phosphate pathway, which is activated for the degradation of galactose and lactose in some bacteria, such as *Staphylococcus aureus* and *Streptococcus lactis* [6]. Moreover, tagatose was generally recognized as safe by the Food and Drug Administration in the USA, because it has no negative impacts on human health, and it is currently used in the food industry as a low-calorie sweetener, due to its low caloric content (1.5 kcal/g) and reduced glycemic index compared to sucrose [5,7].

Due to its negligible effects on human health and the environment, tagatose has attracted the attention of the agricultural sector and showed promising efficacy in suppressing plant diseases caused by a wide range of phytopathogens, such as potato and tomato late blight (*Phytophthora infestans*), grapevine downy mildew (*Plasmopara viticola*), grapevine powdery mildew (*Erysiphe necator*) and cabbage downy mildew (*Hyaloperonospora parasitica*) [8,9,10,11,12,13,14,15,16,17]. In particular, tagatose inhibited *P. infestans* growth in vitro [8,9,10,12] and reduces late blight symptoms on tomato plants under greenhouse conditions [14,15]. Tagatose inhibits sugar metabolism and mitochondrial processes of *P. infestans* [8,10,12], and it can activate grapevine resistance against downy mildew [16,17], suggesting multiple mechanisms of action against phytopathogens. In particular, tagatose causes severe mitochondrial alterations in *P. infestans*, with the consequent decrease in ATP content, accumulation of reactive oxygen species, and downregulation of genes involved in transport, sugar metabolism, signal transduction, and growth-related processes [8,10]. In addition to testing pure tagatose on its own for disease control, efforts have been made to test its efficacy in mixtures with other compounds [16,17]. Herein, we refer to any chemical mixture that contains tagatose as a formulation. Although tagatose efficacy against tomato late blight was largely documented under greenhouse conditions [14,15], no information is available on tagatose persistence on tomato leaves. Considering the high solubility of tagatose in water [18], decreasing efficacy against phytopathogens is expected under field conditions in case of heavy rain or prolonged rainy periods [19]. Moreover, plant leaves are usually colonized by a variety of microorganisms [20,21,22], but no information is available on the possible presence of tagatose-metabolizing bacteria on tomato leaves. For example, tagatose can be used as a carbohydrate source by only certain microbial taxa, such as some isolates that belong to *Erwinia* sp., *Exiguobacterium* sp., *Lactobacillus* sp., and *Lactococcus* sp. [23,24,25,26,27]. Moreover, tagatose can act as a nutritional factor for some bacteria associated with grapevine leaves (e.g., *Chroococcidiopsis* sp., *Erwinia* sp., *Exiguobacterium* sp., *Leifsonia* sp., *Methylobacterium* sp., *Pelomonas* sp., *Pseudomonas* sp. and *Rhodobium* sp.) [13], but the possible effect of phyllosphere microorganisms on tagatose persistence on plant leaves is unknown. The aim of this study was to evaluate the persistence of pure tagatose (TAG) and a formulated product that contained tagatose (tagatose formulation; F_TAG) on tomato leaves under greenhouse conditions to provide better knowledge of this fungal inhibitor. 

## 2. Results

### 2.1. Tagatose Persistence on Tomato Leaves 

**Figure 1:**
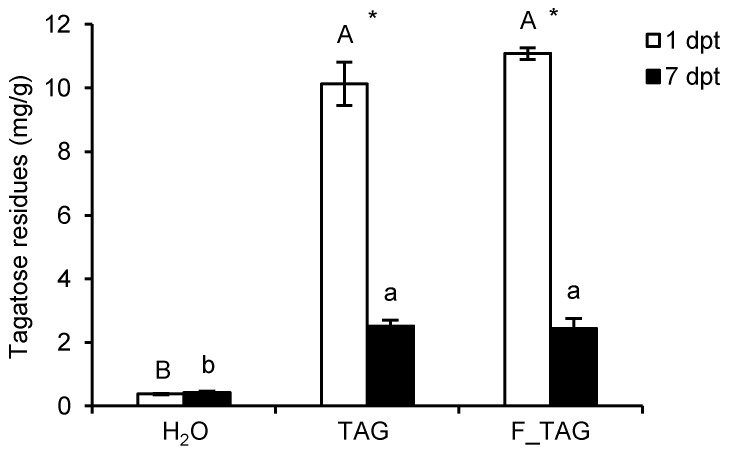
Tagatose residues on tomato leaves. Tomato plants were treated with water (H_2_O), 5 g/L pure tagatose (TAG), or a formulated product that contained 5 g/L tagatose (F_TAG). Tagatose was assessed in leaf washing suspensions of H_2_O-treated, TAG-treated, and F_TAG-treated leaves collected at one (white bars) and seven (black bars) days post-treatment (dpt) and expressed per unit of leaf fresh weight (mg/g). The two-way analysis of variance (two-way ANOVA) showed no significant differences between the two experimental repetitions (*p* > 0.05, three replicates per experiment), and data from the two experiments were pooled. Mean and standard error values of six replicates (each as a pool of 50 leaves collected from 20 plants) from the two experiments are presented for each treatment. Different uppercase and lowercase letters indicate significant differences among treatments at 1 dpt and 7 dpt, respectively, according to Tukey’s test (*p* ≤ 0.05). For each treatment, significant differences between 1 dpt and 7 dpt are marked with an asterisk (*), according to the Student’s *t*-test (*p* ≤ 0.05).

Tomato plants were grown under commercial greenhouse conditions and treated with 5 g/L TAG or a formulated product that contained 5 g/L tagatose (tagatose formulation; F_TAG). The amount of tagatose laid on tomato leaves was assessed at one and seven days post-treatment (dpt), and it was higher in leaf washing suspensions of TAG-treated and F_TAG-treated plants, compared to H_2_O-treated plants (Figure 1). A negligible amount of tagatose was found on H_2_O-treated plants, with no differences between 1 dpt and 7 dpt. Tagatose residues on leaves of TAG-treated and F_TAG-treated plants decreased from 1 dpt to 7 dpt under greenhouse conditions, with a reduction of 75.22 ± 1.96% and 78.01 ± 2.71% of tagatose content in the leaf washing suspensions, respectively. 

**Figure 2:**
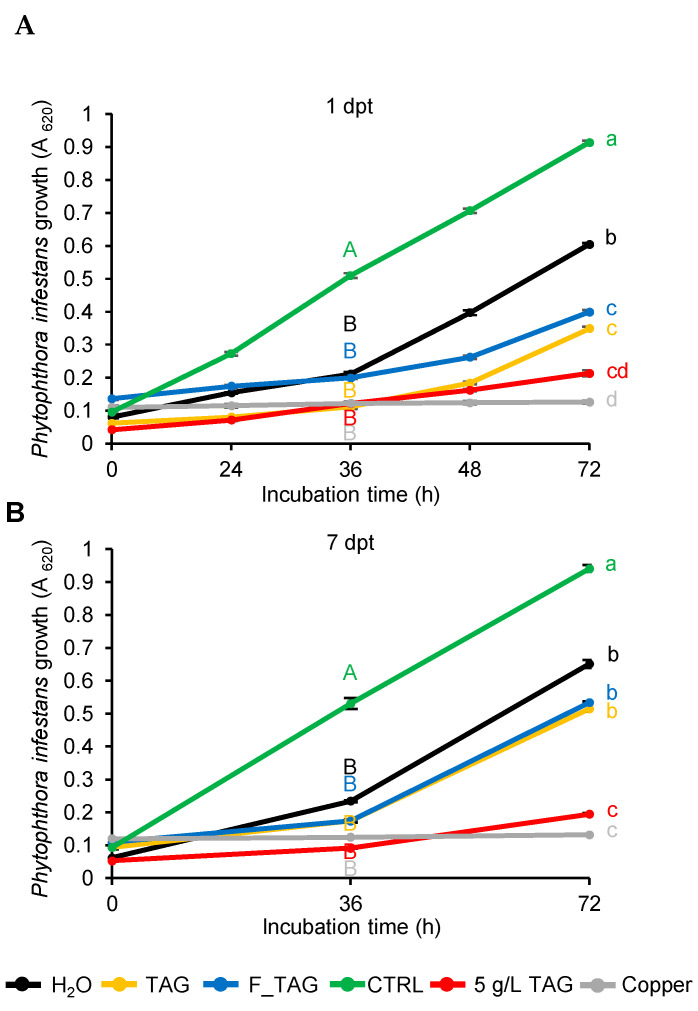
*Phytophthora infestans* growth in the presence of leaf washing suspensions. *Phytophthora infestans* sporangia were incubated in the presence of leaf washing suspensions collected at one (**A**) and seven (**B**) days post-treatment (dpt) from plants treated with water (H_2_O; black), 5 g/L pure tagatose (TAG; yellow) or a formulated product that contained 5 g/L tagatose (F_TAG; blue). Sporangia were incubated in water (CTRL; green), tagatose (5 g/L; red), and copper hydroxide (10 g/L; grey) as controls. *P. infestans* growth was assessed by measuring the absorbance at 620 nm (A_620_) at 0, 36, and 72 h of incubation. The two-way analysis of variance (two-way ANOVA) showed no significant differences between the two experimental repetitions (*p* > 0.05, three replicates per experiment), and data from the two experiments were pooled. Mean and standard error values of six replicates (wells) from the two experiments are presented for each treatment. Different uppercase and lowercase letters indicate significant differences among treatments at 36 and 72 h of incubation, according to Tukey’s test (*p* ≤ 0.05). No differences were found among treatments at 0 h of incubation, according to Tukey’s test (*p* > 0.05).

**Figure 3:**
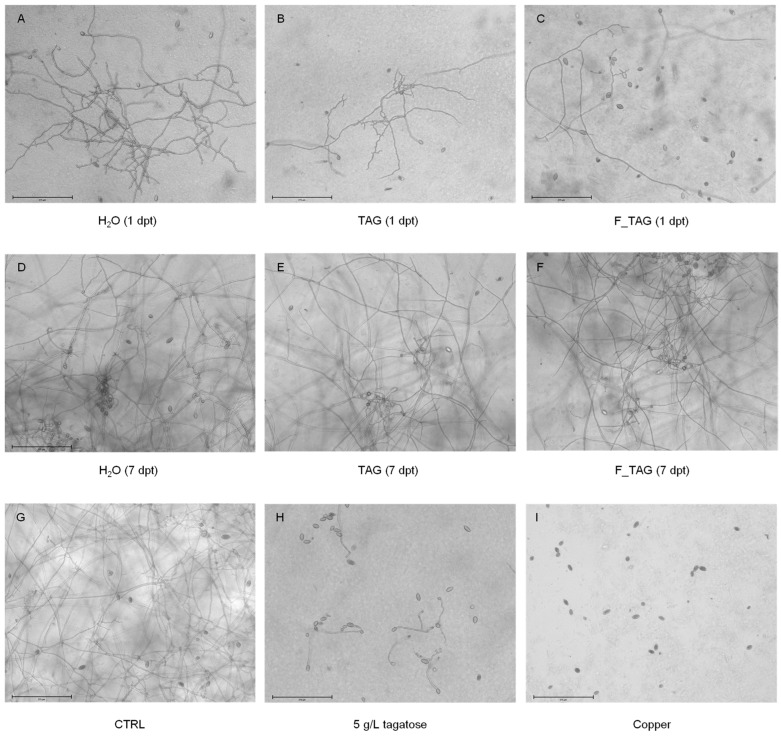
Pictures of *Phytophthora infestans* growth inhibition by leaf washing suspensions. *Phytophthora infestans* sporangia were incubated in the presence of leaf washing suspensions collected at one and seven days post-treatment (dpt) from plants treated with water (H_2_O; (**A**,**D**)), 5 g/L pure tagatose (TAG; (**B**,**E**)) or a formulated product that contained 5 g/L tagatose (F_TAG; (**C**,**F**)). Sporangia were incubated in water (CTRL; **G**), tagatose (5 g/L, **H**), and copper hydroxide (10 g/L; **I**) as controls. Representative pictures were taken at 72 h of incubation. Bars correspond to 275 µm.

To evaluate the inhibitory effects of tagatose residues against *P. infestans*, a sporangia suspension was incubated in pea broth (PB) in the presence of leaf-washing suspensions (Figure 2)*. Phytophthora infestans* growth was reduced in the presence of leaf washing suspensions compared to the control (PB supplemented with water) at 36 h and 72 h after incubation. At 72 h after incubation in PB, *P. infestans* growth was lower in the presence of leaf washing suspensions of TAG-treated and F_TAG-treated plants compared to H_2_O-treated plants collected at 1 dpt (Figure 2A), possibly due to the presence of TAG residues. Conversely, *P. infestans* growth was comparable in the presence of leaf washing suspensions of H_2_O-treated, TAG-treated, and F_TAG-treated plants collected at 7 dpt (Figure 2B), corroborating the reduction in tagatose residues on tomato leaves from 1 dpt to 7 dpt. Microscopic observations confirmed the inhibition of *P. infestans* growth in the presence of leaf washing suspensions of TAG-treated and F_TAG-treated plants collected at 1 dpt, but not at 7 dpt (Figure 3). Moreover, *P. infestans* was almost impaired by 5 g/L TAG or 10 g/L copper hydroxide that were used as controls during the incubation assay (Figure 2 and Figure 3).

### 2.2. Potential Tagatose-Degrading Bacteria of Tomato Phyllosphere

**Figure 4:**
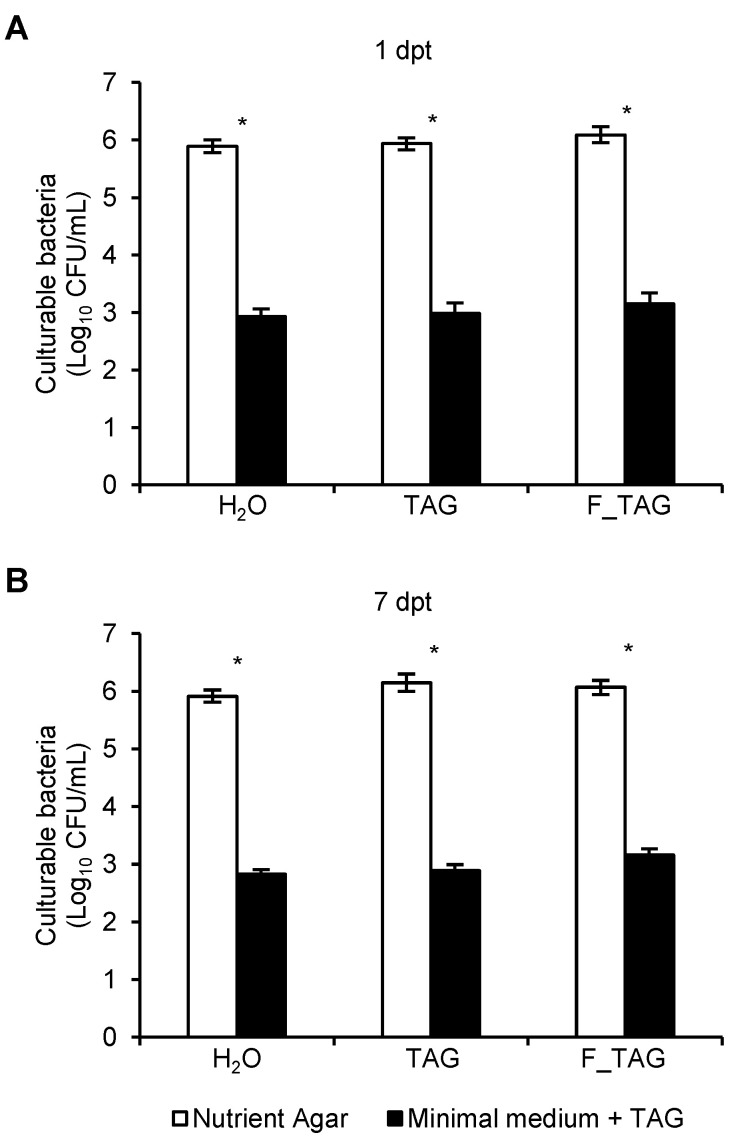
Effects of plant treatments on culturable bacteria of tomato leaves. Bacterial colony-forming units (CFUs) were isolated on nutrient agar (white bars) and a minimal agar medium amended with tagatose, used as the carbon source, (black bars) from leaf washing suspensions collected at one (**A**) and seven (**B**) days post-treatment (dpt) from plants treated with water (H_2_O), 5 g/L pure tagatose (TAG) or a formulated product that contained 5 g/L tagatose (F_TAG). The two-way analysis of variance (two-way ANOVA) showed no significant differences between the two experimental repetitions (*p* > 0.05, three replicates per experiment), and data from the two experiments were pooled. Mean and standard error values (Log_10_-transformed) from six replicates (each as a pool of 50 leaves collected from 20 plants) from the two experiments are presented for each treatment. For each treatment, significant differences between bacterial CFUs on nutrient agar and a minimal agar medium amended with tagatose are marked with an asterisk (*), according to the Student’s *t*-test (*p* ≤ 0.05). No differences were found among treatments at 1 dpt and 7 dpt, according to Tukey’s test (*p* > 0.05).

Due to the reduction in tagatose residues on tomato leaves in the absence of rain and wash-off risks under greenhouse conditions, the presence of potential tagatose-degrading bacteria in the tomato phyllosphere was investigated. Comparable numbers of bacterial colony-forming units (CFUs) grown on nutrient agar (NA) were found in leaf washing suspensions of H_2_O-treated, TAG-treated, and F_TAG-treated plants collected at 1 dpt or 7 dpt (Figure 4). The number of CFUs obtained from tomato leaves was lower on the minimal agar medium amended with 5 g/L tagatose as the carbon source (potential tagatose-degrading bacteria), compared to the NA medium (total culturable bacteria). However, CFU counts on the minimal agar medium amended with tagatose were comparable in H_2_O-treated, TAG-treated, and F_TAG-treated plants collected at 1 dpt or 7 dpt (Figure 4), indicating no enrichment of potential tagatose-degrading bacteria on TAG-treated and F_TAG-treated leaves. 

**Figure 5:**
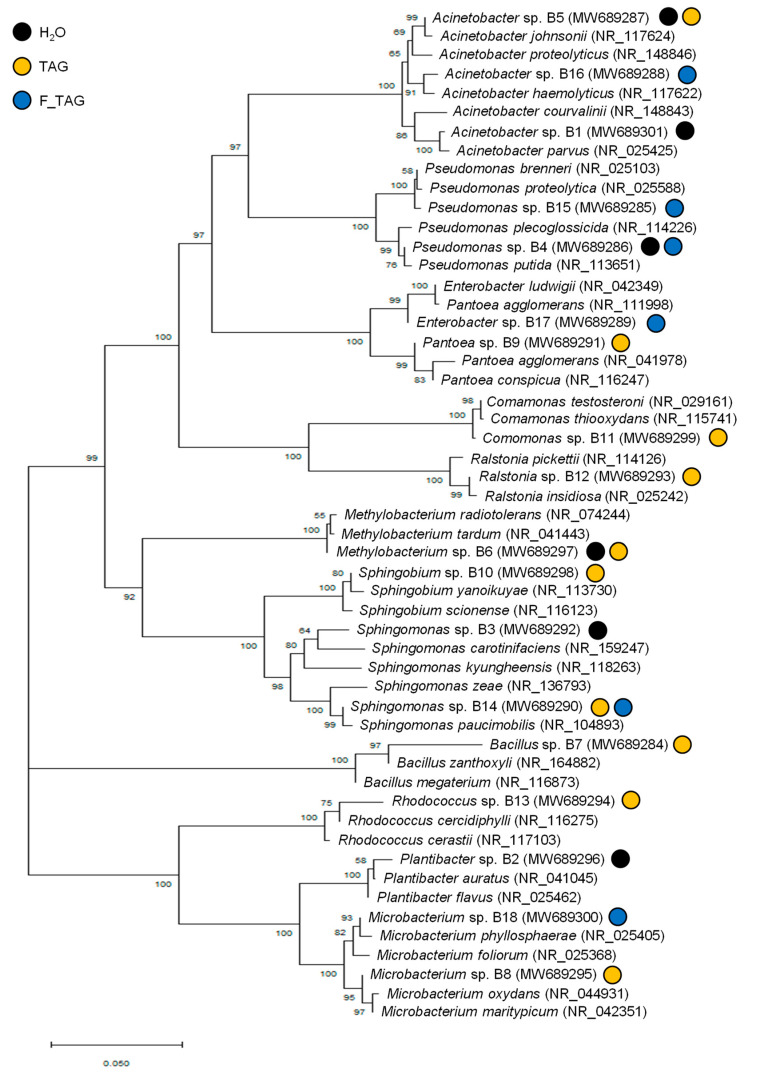
Neighbor-joining phylogenetic tree of potential tagatose-degrading bacteria. Bacterial isolates were obtained from leaf washing suspensions of tomato plants treated with water (H_2_O; black), 5 g/L pure tagatose (TAG; yellow), or a formulated product that contained 5 g/L tagatose (F_TAG; blue) by incubation on a minimal agar medium amended with 5 g/L tagatose as the carbon source (potential tagatose-degrading bacteria; Table 1). The analysis was conducted with Kimura’s two-parameter calculation model in MEGA version 6.0, using the two best BLAST hits of 16S rRNA gene sequences at NCBI. Bootstrap values out of 1000 replicates are shown at the nodes and the scale bar represents the number of substitutions per site.

**Figure 6:**
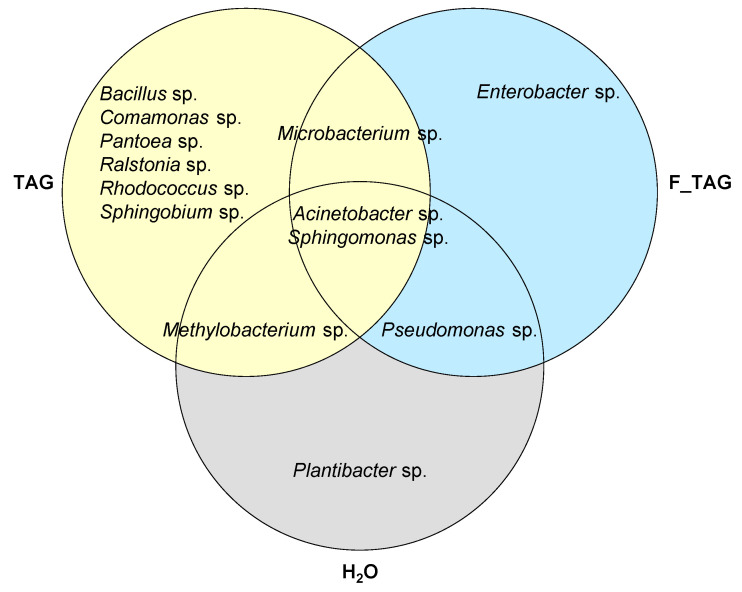
Venn diagram of potential tagatose-degrading bacterial genera isolated from tomato leaves. Bacterial isolates were obtained from leaf washing suspensions of tomato plants treated with water (H_2_O), 5 g/L pure tagatose (TAG), or a formulated product that contained 5 g/L tagatose (F_TAG) by incubation on a minimal agar medium amended with 5 g/L tagatose as the carbon source (potential tagatose-degrading bacteria; Table 1). The Venn diagram indicates the distribution of potential tagatose-degrading bacterial genera among samples.

Representative bacterial isolates were selected visually from each treatment based on morphological analysis of bacterial colonies grown on the minimal agar medium amended with tagatose (Figure 5 and Table 1). The taxonomic annotation was carried out on representative isolates of potential tagatose-degrading bacteria; *Plantibacter* sp. was isolated from H_2_O-treated plants; *Bacillus* sp., *Comamonas* sp., *Pantoea* sp., *Ralstonia* sp., *Rhodococcus* sp. and *Sphingobium* sp. were isolated from TAG-treated plants; and *Enterobacter* sp. from F_TAG-treated plants. The genera *Acinetobacter* and *Sphingomonas* were found as potential tagatose-degrading bacteria in the leaf washing suspensions of all the three treatments (H_2_O, TAG, and F-TAG), while *Methylobacterium*, *Microbacterium*, and *Pseudomonas* were found in the case of two of the three treatments (Figure 6).

## 3. Discussion

Tagatose is known to suppress late blight symptoms on tomato plants under greenhouse conditions [14,15] and to inhibit *P. infestans* growth in vitro [8,9,10,12]. A negligible amount of tagatose was found on H_2_O-treated plants in our experiments, and it was also previously detected on untreated cucumber leaves [11] and grapevine leaves [17], as well as in apples, pineapples, oranges, and raisins [5], suggesting that a small tagatose quantity could be produced by plant and/or microbial metabolism. Moreover, the amount of tagatose decreased after spray application on tomato leaves from 1 dpt to 7 dpt on TAG-treated and F_TAG-treated plants under commercial greenhouse conditions, in the absence of rain wash-off. Although additional time points, time zero included, should be analyzed to identify the kinetics of decreasing tagatose, our results agreed with previous findings on the 7-day residual effects of tagatose against cucumber downy mildew [15]. The application of tagatose in a formulation with other compounds can increase the efficacy against grapevine downy mildew [16,17], cucumber downy mildew, and cucumber powdery mildew [11]. In particular, the use of this formulation can improve tagatose penetration by cucumber roots [11], the upregulation of defense-related genes, and the accumulation of stilbene phytoalexins in grapevine plants [16,17]. Although the co-formulants included in F_TAG are protected by an industrial secret and cannot be tested separately, comparable amounts of tagatose residues and similar inhibitory effects were found in leaf washing suspensions of TAG-treated and F_TAG-treated tomato plants, suggesting no relevant improvement in tagatose persistence by the co-formulants included in the F_TAG product. Thus, further analyses with novel co-formulants or combined applications with other fungicides are required to improve tagatose persistence on tomato leaves. Different co-formulants have been proposed for tagatose, including non-ionic, anionic, cationic, and amphoteric surfactants, water-soluble polymers, amino acids, amino sugars, disaccharide alcohols, and salts [14]. Moreover, clays (<2 mm particle size fraction of montmorillonite or kaolinite) have been proposed to improve the resistance of insecticidal proteins produced by *Bacillus thuringiensis* against microbial degradation [28], suggesting that optimized clay formulations can be tested to limit microbial degradation of tagatose on plant leaves.

Plant leaves harbor complex microbial communities, including many different genera of bacteria, filamentous fungi, yeasts, algae, and protozoa [20,21,22]. Bacteria are the most abundant inhabitants of the phyllosphere [20] and leaf-associated bacterial communities of tomato are known to be dominated by *Acinetobacter* sp., *Methylobacterium* sp., *Pseudomonas* sp., and *Sphingomonas* sp. [29,30]. However, tagatose is not catabolized by most of the bacterial taxa [25] and the number of potential tagatose-degrading bacteria was lower compared to the number of total culturable bacteria in the tomato leaf samples analyzed in this study. The concentration of tagatose (5 g/L) used in the minimal agar medium was within the range of concentrations (4–20 g/L) commonly used for the assessment of carbon source utilization by bacterial isolates [31,32,33]. However, the lack of an osmotic control in bacterial isolation suggests that culturable bacteria isolated from tomato leaves on the minimal agar medium amended with tagatose can also display tolerance to osmotic stress, provided by this sugar concentration, and further analyses are required to better assess this bacterial tolerance. In particular, these bacteria isolates belonged to *Acinetobacter* sp., *Bacillus* sp., *Comamonas* sp., *Enterobacter* sp., *Methylobacterium* sp., *Microbacterium* sp., *Pantoea* sp., *Plantibacter* sp., *Pseudomonas* sp., *Ralstonia* sp., *Rhodococcus* sp., *Sphingobium* sp., and *Sphingomonas* sp. Although further metabolic studies are required to confirm the use of tagatose as a carbon source, these isolates of tomato phyllosphere are potential tagatose-degrading bacteria that can affect tagatose persistence on tomato leaves. Tagatose treatments are known to increase the relative abundance of *Methylobacterium* sp. and *Pseudomonas* sp. on grapevine leaves [13], indicating that tagatose can act as a nutritional factor for some phyllosphere bacteria hosted by different plant species. Interestingly, tagatose is also known to be metabolized by some human- and food-associated species that belong to *Erwinia*, *Exiguobacterium*, *Lactobacillus*, and *Lactococcus* genera [23,24,25,26,27], indicating the ubiquitous presence of a limited number of taxa that are able to degrade this rare sugar. For example, tagatose can be transported into the cell by phosphotransferase uptake systems and used as an intermediate in the lactose, galactose, and galactitol catabolism by some bacterial species [25]. In particular, the *Lactobacillus* sp. metabolism includes the tagatose-6-phosphate pathway [23,26], while *Erwinia persicinus* [27] and *Exiguobacterium aurantiacum* [24] can produce acid from tagatose fermentation. Moreover, the ribose isomerase of *Acinetobacter* sp. can isomerize tagatose [34], and the tagatose 3-epimerase of *Pseudomonas* sp. can convert tagatose to fructose [35]. Thus, further metataxonomic (16S amplicon sequencing) and shotgun metagenomic studies are required to better understand the whole taxonomic composition and to investigate possible metabolic traits (e.g., genes encoding tagatose-degrading enzymes) of phyllosphere microbial communities of tomato plants. Moreover, the tagatose persistence on surface-sterilized leaves or in vitro-grown plants should be assessed to verify tagatose persistence in the absence of phyllosphere microorganisms and to better quantify the contribution of phyllosphere bacteria on tagatose degradation, after spray application on tomato plants.

## 4. Materials and Methods

### 4.1. Biological Material and Growth Conditions

Tomato plants (*Solanum lycopersicum* variety Moneymaker) were grown for two months under commercial greenhouse conditions (plastic tunnel) at 25 ± 5 °C and 60 ± 10% relative humidity.

*Phytophthora infestans* strain VB3 was stored in glycerol at −80 °C in the fungal collection of the Fondazione Edmund Mach and it is freely available upon request [8,10,12]. *Phytophthora infestans* was grown in Petri dishes on pea agar medium (PAM, 12.5% frozen peas, and 1.2% agar in distilled water) at 18 ± 1 °C [10]. To collect *P. infestans* sporangia, seven-day-old *P. infestans* dishes were filled with 2 mL pea broth (PB, 12.5% frozen peas in distilled water). Sporangia were then scraped with a sterile spatula and the suspension was filtered using a sterile Pasteur pipette that contained a fine mesh. The concentration of the sporangial suspension was assessed by counting with a hemocytometer and it was adjusted to 1 × 10^4^ sporangia/mL. 

### 4.2. Plant Treatments and Collection of Leaf Washing Suspensions

Plants were treated with 5 g/L pure TAG or a formulated product that contained 5 g/L tagatose (F_TAG; wettable powder containing 80% tagatose w/w; IFP48 (MCF1309); Kagawa University, Mitsui Chemicals Agro and Belchim Crop Protection Londerzeel, Belgium). Tagatose concentration (5 g/L) was optimized against *P. infestans* in vitro [8,10,12] and in preliminary experiments on tomato plants (data not shown). As a control, plants were treated with distilled water (H_2_O). Treatments were applied to all leaves using a compressed air hand sprayer (20–30 mL for each plant). Three replicates (pool of 20 plants each) were analyzed for each treatment and the experiment was carried out twice.

Leaf washing suspensions were obtained from H_2_O-treated, TAG-treated, and F_TAG-treated plants, as previously described [36]. Briefly, asymptomatic leaves were randomly collected at 1 dpt and 7 dpt, and 3 replicates of 50 healthy leaves were obtained from 20 plants for each treatment and each experiment. Leaves of each replicate were placed in sterile plastic boxes and washed with 100 mL sterile isotonic solution (0.85% sodium chloride with 0.01% Tween 20) by orbital shaking at 80 rpm for 15 min. Each leaf washing suspension was filtered with sterile cheesecloth and an aliquot of 50 mL was used for bacterial isolation. The remaining part (50 mL) of each leaf washing suspension was filter sterilized and stored at −20 °C for tagatose quantification and efficacy tests against *P. infestans*. 

### 4.3. Tagatose Quantification

Tagatose content was quantified in each leaf washing suspension (50 mL) that resulted from H_2_O-treated, TAG-treated, and F_TAG-treated plants by ion chromatography (Chemistry Unit at Fondazione Edmund Mach), as previously reported [13], and it was converted to tagatose residues per unit of fresh leaf weight (mg/g), using a calibration curve of 98.5% pure tagatose (Sigma-Aldrich, Merck, Kenilworth, NJ, USA) dissolved in ultrapure water, within a range between 2 and 40 μg/mL. Briefly, samples were diluted 50-fold in ultrapure water, filtered through a 0.45 μm PTFE membrane (Sartorius, Goettingen, Germany), and analyzed with an ionic chromatograph ICS 5000 (Dionex, Thermo Scientific, Waltham, MA, USA), equipped with an autosampler, a quaternary gradient pump, a column oven and a pulsed amperometric detector with a gold working electrode and a palladium counter electrode. The separation was obtained by injecting 5 μL of diluted sample into a CarboPac PA200 3 × 250 mm analytical column (Dionex, Thermo Fisher Scientific, Waltham, MA, USA), preceded by a CarboPac PA200 3 × 50 mm guard column (Dionex, Thermo Fisher Scientific, Waltham, MA, USA), with a KOH gradient (from 1 to 100 mM) at 0.4 mL/min flow rate.

### 4.4. Efficacy Test of Leaf Washing Suspensions against Phytophthora Infestans Growth

The sporangial suspension of *P. infestans* (1 × 10^4^ sporangia/mL) was prepared as described above and incubated in PB medium (90 µL) in the presence of an aliquot (100 µL) of leaf washing suspension, obtained from H_2_O-treated, TAG-treated and F_TAG-treated plants, in a 96-well microplate. As controls, the sporangia suspension was incubated in distilled water (control, CTRL), pure tagatose (5 g/L), and copper hydroxide (10 g/L, Sigma-Aldrich, Merck). *Phytophthora infestans* growth was assessed by measuring the absorbance at 620 nm using a TECAN Microplate Reader (Infinite F200 Pro Luminometer, Männedorf, Switzerland) at 0, 36, and 72 h after incubation at 18 ± 1 °C, under orbital shaking at 80 rpm. Three replicates (wells) were assessed for each treatment and the experiment was carried out twice. Microscopic observations of *P. infestans* growth were carried out at 72 h with an EVOS FL Auto Imaging System (Thermo Fisher Scientific, Waltham, MA, USA).

### 4.5. Isolation of Culturable Bacteria 

To quantify total culturable bacteria, 1 mL of each leaf washing suspension was 10-fold serially diluted and 0.1 mL of each dilution was plated on NA medium (1 g/L meat extract, 5 g/L peptone, 5 g/L sodium chloride, 2 g/L yeast extract, and 15 g/L agar; Sigma-Aldrich, Merck). A minimal agar medium was prepared (0.4 g/L K_2_HPO_4_, 0.4 g/L KH_2_PO_4_, 0.4 g/L (NH_4_)_2_PO_4_, 0.3 g/L NaCl, micronutrients (200 mg/L FeSO_4_·7H_2_O, 10 mg/L ZnSO_4_·7H_2_O, 3 mg/L MnCl_2_·4H_2_O, 20 mg/L CoCl_2_·6H_2_O, 1 mg/L CuCl_2_·2H_2_O, 2 mg/L NiCl_2_·6H_2_O, 500 mg/L Na_2_MoO_4_·2H_2_O and 30 mg/L H_3_BO_3_) and 15 g/L agar) [37] and amended with 5 g/L tagatose (filter sterilized stock solution of 50 g/L tagatose) as the carbon source (within the range of concentrations from 4 to 20 g/L used for the assessment of carbon source utilization by bacterial isolates [31,32,33]), in order to isolate potential tagatose-degrading bacteria. After incubation at 25 °C for 48 h, CFU counts per mL of leaf washing suspension were determined. Representative bacterial isolates were selected visually for each treatment based on morphological analysis of bacterial colonies (i.e., size, color, opacity, texture, form, elevation, and margin) [36,38] grown on the minimal agar medium, amended with tagatose as the carbon source (potential tagatose-degrading bacteria), in order to collect the whole biodiversity and avoid redundances based on colony morphology. 

### 4.6. Identification of Bacterial Isolates

The identification of bacterial isolates was carried out by 16S rRNA gene sequencing, as described by Esmaeel et al. [39]. Briefly, each isolate was grown in LB broth (10 g/L tryptone, 5 g/L yeast extract, 5 g/L NaCl, and pH 7.2) at 28 °C for 18 h, under orbital shaking at 160 rpm. Total DNA was extracted using the Wizard Genomic Purification DNA Kit (Promega Corp., Madison, WI, USA), and the 16S rRNA gene fragment was amplified with FD2 (5′-AGAGTTTGATCATGGCTCAG-3′) and RP1 (5′-ACGGTTACCTTGTTACGACTT-3′) primers, using a PCR Master Mix (Fermentas, Thermo Fisher Scientific, Waltham, MA, USA) with 5 µL of DNA (10 to 40 ng/µL) in 50 µL with the following amplification protocol: 94 °C for 5 min, 30 cycles at 94 °C for 1 min, 56 °C for 30 s and 72 °C for 2 min, and a final extension at 72 °C for 10 min. The resulting PCR product (from 1450 to 1500 bp) was purified by gel electrophoresis, followed by the GeneJET Gel Extraction Kit (Fermentas, Thermo Fisher Scientific, Waltham, MA USA) and sequenced in both directions by Sanger sequencing technology at Genewiz (Leipzig, Germany).

After quality trimming, forward and reverse sequences were aligned and merged to obtain a consensus sequence. Each sequence was aligned with the nucleotide basic local alignment search tool (BLASTn) at the National Center for Biotechnology Information (NCBI, 16S rRNA database; accessed on 21 February 2022) for taxonomic annotation and the two best BLAST hits of 16S rRNA gene sequences were downloaded for phylogenetic analysis. The phylogenetic tree was constructed by the neighbor-joining method, with Kimura’s two-parameter calculation model in MEGA version 6.0 (Molecular Evolutionary Genetics Analysis, Pennsylvania State University, State College, PA, USA; https://www.megasoftware.net/) [40]. The evolutionary distances were computed using the maximum composite likelihood method [41]. The rate variation among sites was modeled with a gamma distribution with five rate categories. Bootstrap analysis with 1000 replicates was performed to assess the support of the clusters [42]. Although potential functions can be only partially hypothesized by taxonomic identification at the genus level, bacterial isolates were classified as potential biocontrol agents of tomato pathogens and as potential growth promoters of tomato plants, according to a literature search on the functional properties of the comprised species. All 16S sequences were submitted to the NCBI database (accession numbers from MW689284 to MW689301).

### 4.7. Statistical Analysis

All experiments were carried out twice and the data were analyzed with Statistica 13.1 software (Tibco, Palo Alto, CA, USA). Normal distribution (Kolmogorov–Smirnov test, *p* > 0.05) and variance homogeneity of the data (Levene’s tests, *p* >0.05) were checked, and parametric tests were used when both assumptions were respected. Each experimental repetition was analyzed singularly, and factorial analysis of variance (factorial ANOVA) was used to demonstrate non-significant differences between the two experiments (*p* > 0.05). Data from the two experimental repetitions were pooled and significant differences were assessed with the Student’s *t*-test (*p* ≤ 0.05) or Tukey’s test (*p* ≤ 0.05) in case of pairwise or multiple comparisons, respectively. 

## 5. Conclusions

Tagatose is a safe molecule proposed for the sustainable control of tomato late blight. However, the amount of tagatose residues on tomato leaves and the inhibitory activity against *P. infestans* decreased after spray application under greenhouse conditions, even in the absence of rain. Our data report the presence of potential tagatose-degrading bacteria on tomato leaves, indicating that indigenous phyllosphere microorganisms could partially metabolize the tagatose laid on plant leaves after spray application, reducing the persistence of this fungal inhibitor. Further studies on formulations and combinations with other plant protection products are required to improve tagatose persistence under greenhouse and field conditions.

## Figures and Tables

**Table 1:** List of potential tagatose-degrading bacteria isolated from leaf washing suspensions.

Accession Number ^1^	Isolate Code ^2^	Plant Treatment ^3^	Taxonomic Identification ^4^	BLAST Best Hit ^5^	Sequence Identity (%) ^6^
MW689301	B1	H_2_O	*Acinetobacter* sp.	*Acinetobacter parvus*	99.25
MW689296	B2	H_2_O	*Plantibacter* sp.	*Plantibacter flavus*	99.58
MW689292	B3	H_2_O	*Sphingomonas* sp.	*Sphingomonas kyungheensis*	96.75
MW689286	B4	H_2_O and F_TAG	*Pseudomonas* sp.	*Pseudomonas putida*	99.90
MW689287	B5	H_2_O and TAG	*Acinetobacter* sp.	*Acinetobacter johnsonii*	99.92
MW689297	B6	H_2_O and TAG	*Methylobacterium* sp.	*Methylobacterium radiotolerans*	99.92
MW689284	B7	TAG	*Bacillus* sp.	*Bacillus megaterium*	99.88
MW689295	B8	TAG	*Microbacterium* sp.	*Microbacterium oxydans*	99.46
MW689291	B9	TAG	*Pantoea* sp.	*Pantoea agglomerans*	98.88
MW689298	B10	TAG	*Sphingobium* sp.	*Sphingobium yanoikuyae*	99.69
MW689299	B11	TAG	*Comamonas* sp.	*Comamonas testosteroni*	99.39
MW689293	B12	TAG	*Ralstonia* sp.	*Ralstonia insidiosa*	99.63
MW689294	B13	TAG	*Rhodococcus* sp.	*Rhodococcus cercidiphylli*	99.59
MW689290	B14	TAG and F_TAG	*Sphingomonas* sp.	*Sphingomonas paucimobilis*	99.71
MW689285	B15	F_TAG	*Pseudomonas* sp.	*Pseudomonas brenneri*	99.71
MW689288	B16	F_TAG	*Acinetobacter* sp.	*Acinetobacter haemolyticus*	98.94
MW689289	B17	F_TAG	*Enterobacter* sp.	*Enterobacter ludwigii*	99.54
MW689300	B18	F_TAG	*Microbacterium* sp.	*Microbacterium phyllosphaerae*	99.62

^1^ Accession numbers of 16S sequences submitted at the National Center for Biotechnology Information (NCBI) of potential tagatose-degrading bacteria isolated from leaf washing suspensions of tomato plants by incubation on a minimal agar medium amended with 5 g/L tagatose as the carbon source. ^2^ Representative bacterial isolates were selected visually, and numerical codes were assigned. ^3^ Bacteria were isolated from leaf washing suspensions of tomato plants treated with sterile water (H_2_O), 5 g/L pure tagatose (TAG), or a formulated product that contained 5 g/L tagatose (F_TAG). ^4^ Taxonomic identification was carried out by BLASTn alignment of the 16S rRNA sequence against the NCBI 16S rRNA database. ^5^ Best hits resulting from the BLASTn alignment of the 16S rRNA sequence against the NCBI 16S rRNA database. ^6^ Percentage of sequence identity of each 16S sequence with the BLAST best hit.

## Data Availability

The 16S sequences are available at the NCBI database (accession numbers MW689284 and MW689301).
